# Addressing the Helper’s and Victim’s Gender Is Crucial in Schoolchildren Resuscitation Training—A Prospective, Educative Interventional Trial

**DOI:** 10.3390/jcm11092384

**Published:** 2022-04-24

**Authors:** Sabine Wingen, Hannes Ecker, Daniel C. Schroeder, Bérénice Bartholme, Bernd W. Böttiger, Wolfgang A. Wetsch

**Affiliations:** 1 University Hospital Cologne, Department of Anaesthesiology and Intensive Care Medicine, University of Cologne, Faculty of Medicine, Kerpener Straße 62, 50937 Cologne, Germany; sabine.wingen@uk-koeln.de (S.W.); hannes.ecker@uk-koeln.de (H.E.); berenice.bartholme@uk-koeln.de (B.B.); bernd.boettiger@uk-koeln.de (B.W.B.); 2 German Resuscitation Council, 89070 Ulm, Germany; 3 FOM University of Applied Sciences, 50678 Cologne, Germany; 4 Department of Anesthesiology and Intensive Care, German Armed Forces Central Hospital of Koblenz, Rübenacher Str. 170, 56072 Koblenz, Germany; daniel.schroeder@uk-koeln.de

**Keywords:** cardiopulmonary resuscitation, out-of-hospital cardiac arrest, education and training, bystander, schoolchildren, KIDS SAVE LIVES

## Abstract

Background: A victim’s gender is a known factor that influences the willingness of adult bystanders to perform cardiopulmonary resuscitation (CPR) if an out-of-hospital cardiac arrest (OHCA) occurs. This study aims to identify whether gender characteristics of OHCA victims are also relevant to schoolchildren, who are the key target group of CPR trainings worldwide. Methods: A prospective, educative intervention study was performed in schoolchildren (5th–7th grade). Schoolchildren’s willingness to perform CPR was assessed by means of questionnaires before (t0) and after (t1) standardized CPR training. Participants were asked how determined they were to perform CPR in male and female OHCA victims on a 5-point Likert scale (not being determined to being very determined). A data analysis was performed according to the gender characteristics of schoolchildren. Results: Overall, 342 schoolchildren aged 10–15 years were included, and 166 male (MG) and 176 female (FG) schoolchildren served as a comparison group. Before (t0) and after (t1) the intervention, females showed a significantly higher general willingness to perform CPR than males (t0: 97.1% vs. 89.0%; *p* < 0.003 and t1: 95.7% vs. 98.9%; *p* = 0.038). The general willingness to perform CPR after training had a stronger increase in males (8.0% vs. 2.3%; *p* = 0.017). In the case that the OHCA victim was female, male schoolchildren were less willing to perform CPR than females at baseline (MG: *n* = 101;60.8% vs. FG: *n* = 147;84.5%; *p* < 0.001) and after training (MG: *n* = 97;58.4% vs. FG: *n* = 138;79.3%; *p* < 0.001). At t1, CPR willingness for female victims was improved in males (MG: *n* = 36;21.7% vs. FG: *n* = 19;10.9%; *p* = 0.006). Conclusions: The gender characteristics of OHCA victims, as well as schoolchildren themselves, have a relevant impact on the willingness to perform CPR. Training concepts should effectively motivate male schoolchildren to reduce preexisting inhibitions, especially towards female OHCA patients. Trial registration: This study was registered at the German Clinical Trials Register (Registration number: DRKS00017707) on 2 August 2019.

## 1. Introduction

Out-of-hospital cardiac arrest (OHCA) has an incidence of 84 out of 100.000 inhabitants per year and reveals a leading cause of death in industrialized nations, resulting in a major public health issue [1]. With a reported survival of only 8%, the prognosis of patients suffering from OHCA is alarmingly poor [1]. The immediate initiation of cardiopulmonary resuscitation (CPR) attempts will increase a patient’s survival by the factor 2–3 [2,3,4]. Therefore, bystanders can successfully reduce the number of deaths, decrease long-term neurological brain damage, and save on health-care and follow-up costs in patients with OHCA [5,6,7]. However, bystander CPR rates vary widely between European countries [4], ranging from a reported 13% to 82% and average of 58%. Public interventions and programs have been implemented to increase public awareness and emphasize the importance of bystander CPR in the case of OHCA [7]. Additionally, the education of bystanders, including schoolchildren in CPR, is recommended by European resuscitation guidelines [8].

Schoolchildren are an important target group for increasing bystander CPR knowledge and skills, as they act as multipliers by passing on their knowledge at home to their family and friends [9,10]. Children are able to adequately perform CPR after appropriate training [11,12,13]. Consequently, since 2015, the World Health Organization has recommended the annual repetition of a two-hour-theoretical and practical CPR training session, starting at the age of 12 years [14]. Following the ‘KIDS SAVE LIVES’ initiative, few countries have implemented mandatory schoolchildren CPR training programs, including Italy, France, Belgium, Denmark, and Portugal [15]. Furthermore, a recommendation for CPR training in schoolchildren exists in an additional 23 European countries [15]. 

Primarily, CPR training programs for schoolchildren focused on knowledge and skills education [14]. Within recent years, soft skills (i.e., attitude, self-confidence) became more important in CPR education [16]. Considering that studies identified significantly lower bystander CPR rates in female OHCA victims, gender aspects in CPR training are now taken into account and are a new focus in this research field. [17,18,19]. It is still unclear, whether the gender of OHCA victims matters in schoolchildren’s willingness to perform bystander CPR. Differences between male and female schoolchildren in this aspect have so far not been studied. Thus, this study aims to identify the relevance of gender characteristics in schoolchildren’s willingness to perform CPR on OHCA victims with different sexes. Furthermore, the extent to which training can change their willingness to provide CPR was studied.

## 2. Materials and Methods

This study is a sub-study of a prospective, randomized, educative interventional study, which was performed from August to November 2019 in a secondary school in Germany. 

### 2.1. Ethical Approval

The study received ethical approval by the Ethics Committee of the University of Cologne (Head: Prof. Dr. Voltz; No. 19-1249).

### 2.2. Trial Registration

This study was registered at the German Clinical Trials Register (Registration number: DRKS00017707) on 2 August 2019.

### 2.3. Study Population

Grade 5 to 7 schoolchildren from one secondary school in Germany were asked to participate in the trial after being provided with comprehensive study information with precise written explanations about the aim of the study, conditions of participation, and data privacy. All data acquired were pseudonymized. Participants under 14 years of age also needed written declaration of consent from their parents. 

### 2.4. Questionnaire

Schoolchildren’s willingness to perform CPR was assessed by means of validated questionnaires before (t0) and immediately after (t1) CPR training. The questionnaires contained three items [20]. First, participants were asked the question: “In the event of a clearly recognizable cardiac arrest (unconsciousness and no breathing), would you resuscitate the victim?” (response category: yes/no), Secondly, participants were asked to answer the questions: “How determined are you to start resuscitation in case the victim is male?” and “How determined are you to start resuscitation in case the victim is female?” (response category: 5-point Likert scale from “not being determined” to “being very determined”). Demographic parameters, such as gender, age, and pre-experience in first aid were also evaluated.

### 2.5. Intervention

One training group received an innovative e-learning CPR training online session, including a simulated CPR training video scenario and a supplemental CPR gaming scenario with theoretical and practical CPR tasks in a first-person view. The second group received analogous conventional face-to-face CPR training divided into theoretical CPR education, presenting slides and a practical hands-on CPR training session with individual resuscitation manikins. Training content was similar in both groups and designed based on European and German CPR education guidelines and curricula [21,22]. The training content was the (i) function of the cardiovascular system, (ii) medical background to and epidemiology of OHCA, (iii) information about bystander CPR rates, (iv) detection of an OHCA, (v) emergency call, and (vi) chest compression (hands position, compression frequency and depth). We deliberately chose existing, validated training concepts, which had not yet addressed gender aspects.

### 2.6. Statistical Analysis

**Figure 1:**
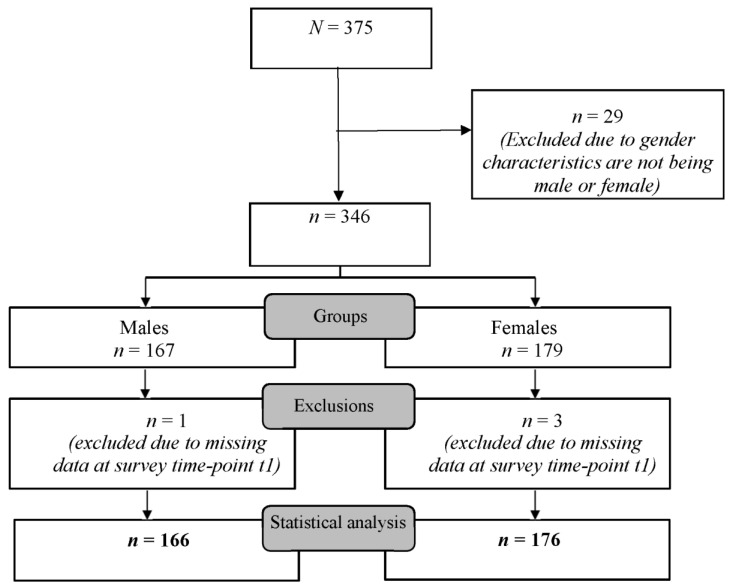
Data extraction sheet.

For statistical analysis, participants were assigned into two groups according to their gender characteristics (male/female), independent of their perceived training method. Participants with other gender characteristics were excluded due to missing statistical power in these gender groups. Participants were also excluded from statistical analysis if they or their parents did not provide written consent of participation. Participants that did not attend both survey time points (t0;t1) were also excluded (Figure 1).

General willingness (yes/no) was defined as improved if participants’ general willingness changed from “no” at t0 to “yes” at t1. Willingness to resuscitate female or male OHCA victims was assumed if participants stated that they were rather willing or very willing (Likert scale 4 or 5 points). The improvement of gender-related willingness after CPR training was defined as having scored one or more points higher in the 5-point Likert scale compared to baseline evaluation. Data analysis was performed with IBM SPSS Statistic Version 26 (IBM Corp., Armonk, NY, USA). Binary data were analyzed using the ×2 test. Ordinary data were analyzed using the Mann–Whitney U test. Statistical significance was accepted as a *p* value of 0.05 or less.

## 3. Results

In total, 375 schoolchildren were included in our study. After removing participants due to pre-defined exclusion criteria, 342 schoolchildren (males *n* = 166; females *n* = 176) were included in the statistical analysis (Figure 1). The demographic characteristics of participants are presented in Table 1.

Differences regarding age (*p* = 0.531), pre-experience in first aid (*p* = 0.564), and perceived CPR training methods of schoolchildren (*p* = 0.833) could not be detected between both groups.

### 3.1. Influence of Schoolchildren’s Gender on Their CPR Willingness 

Our study reveals differences between male and female schoolchildren in the willingness to perform CPR. At baseline, females were more willing to perform CPR on an OHCA victim compared to males (97.1% vs. 89.0%; *p* = 0.011). The general willingness to resuscitate after training was still significantly higher in the female group (*p* = 0.038), but males showed a stronger improvement in their willingness compared to females (MG:8.0% vs. FG: 2.3%; *p* = 0.017) (Table 2).

### 3.2. Influence of OHCA Victims’ Gender on the Willingness to Perform CPR in Schoolchildren before and after CPR Training

Before CPR training, determination to perform CPR on a male OHCA victim was similar between male and female schoolchildren (*n* = 121; 72.9% vs. *n* = 120; 69.0% *p* = 0.409). The situation remained unchanged after CPR training (*n* = 109; 65.7% vs. *n* = 120; 69.0%; *p* = 0.318). Additionally, the improvement in willingness to resuscitate males was equal in both groups after CPR training (*n* = 28; 16.9% vs. *n* = 33; 18.9%; *p* = 0.652) (Table 3).

In contrast, if the OHCA victim was female, male schoolchildren showed a lower determination to perform CPR than females before intervention (*n* = 101; 60.8% vs. *n* = 147; 84.0%; *p* < 0.001). The same applied for the situation after CPR training (*n* = 97; 58.4% vs. 138; 79.3%; *p* < 0.001). After CPR training, the improvement in the determination to perform CPR on a female OHCA victim was significantly improved in the group of male schoolchildren compared to the female group (*n* = 36; 21.7% vs. *n* = 19; 10.9%; *p* = 0.006). 

## 4. Discussion

This is the first study to evaluate the willingness of schoolchildren to provide CPR depending on the victim’s gender. Whereas previous studies revealed differences in bystanders’ willingness to start CPR depending on the victim’s gender [17], no such data have been reported for the population of schoolchildren so far.

This study reveals three major findings. First, female schoolchildren show higher CPR willingness than males. Thus, the general willingness to perform CPR seems to be affected by schoolchildren’s sex. Second, male schoolchildren are less willing to perform CPR if the OHCA victim is female. Third, CPR training reduces reservations about CPR willingness in general, especially for resuscitating women in male schoolchildren.

Gender differences in training outcomes, such as CPR willingness, are scientifically described. A higher willingness to perform bystander CPR in female schoolchildren is a study finding that is supported by Finke et al., who described a better response to cardiac arrest (CA) in female schoolchildren [18]. Female schoolchildren showed higher motivation to attend CPR-training and a higher multiplier effect. In contrast, male students had higher confidence in CPR proficiency and revealed better practical skills [18]. Moreover, Pivac et al. found gender to be a relevant predictor that significantly influenced knowledge levels after CPR training [23]. They concluded that the way in which schoolchildren were approached with CPR training and the concept in which training is organized may influence gender differences [23].

The result of a lower CPR willingness in male schoolchildren if OHCA occurred in females was in line with results scientifically described by Bloem et al. in adult bystanders. In detail, female victims who suffered an OHCA received less bystander CPR attempts than males in real-life OHCA cases [17]. Furthermore, Blewer et al. identified a gender disparity in the public response to OHCA and delivered bystander CPR that was also linked to survival outcomes [24]. Gender barriers, such as fears of inappropriate sexual harassment, fear of causing injury, and accusations of sexual assault, were reported [19]. Additionally, a lack of public knowledge about the fact that women also suffer OHCA was described [25]. Moreover, a manikin study showed that men removed less clothing from female manikins in the case of an OHCA scenario [26]. This is why Kramer et al. claimed that realistic female patient simulators experienced social differences associated with the care of women suffering from OHCA [26]. Current resuscitation manikins suitable for school classes have a neutral gender or embody a male person [27]. In our study, reasons for performing/not performing CPR on male or female OHCA victims were not assessed. Nevertheless, fears and behaviors related to hurting the victim are potential reasons that are generally reported by CPR studies in a target group of schoolchildren [20]. 

Schoolchildren are an important target group to increase public awareness of OHCA and sustainably increase bystander CPR. Many studies show an increased willingness to perform CPR in schoolchildren after appropriate training [20]. To date, there are many different CPR training approaches for schoolchildren [28,29]. Nevertheless, gender aspects in the conducting of CPR trainings are underestimated [18]. The results of our study indicate that gender aspects are underrepresented in current existing and validated CPR training programs. As lower bystander CPR in female OHCA victims results in lower survival rates [17]; therefore, current CPR training programs should be modified to include gender aspects as soon as possible.

The data interpretation of our study is affected by the fact that our results are based on a theoretical survey and may not represent practical behavior in a real-life cardiac arrest scenario. Despite this limitation, this study is the first to examine the influence of schoolchildren’s sex and OHCA victim’s sex on CPR willingness in schoolchildren. Moreover, we performed a single-center study and all involved schoolchildren belonged to the same secondary school. Thus, it is unknown whether our findings also apply to other schools and settings.

## 5. Conclusions

Gender characteristics of OHCA victims and of schoolchildren themselves have a relevant impact on the willingness to perform CPR. The future design of training programs that consider the influence of gender may help to better motivate male schoolchildren to reduce pre-existing inhibitions, especially towards female OHCA patients.

## Figures and Tables

**Table 1:** Demographic characteristics of participating schoolchildren.

Schoolchildren’s Characteristics	Sum	Male Group	Female Group	*p* -Value
**Sex** *n* = 342	*n* (%) 342 (100.0)	*n* (%) 166 (48.5)	*n* (%) 176 (51.5)	
**Age** Median (IQR25–75) *n* = 342	12 (11–13) *n* (%)	12 (11–13) *n* (%)	12 (11–13) *n* (%)	0.531 ^1^
10	29 (8.5)	12 (7.2)	17 (9.7)	
11	95 (27.8)	54 (32.5)	41 (23.2)	
12	107 (31.3)	48 (28.9)	59 (33.5)	
13	96 (28.1)	43 (25.9)	53 (30.1)	
14	14 (4.1)	8 (4.8)	6 (3.4)	
15	1 (0.3)	1 (0.6)	0 (0.0)	
**Pre-knowledge in first aid** *n* = 338				0.564 ^2^
Yes	198 (58.6)	95 (58.3)	103 (58.9)	
No	140 (41.4)	68 (41.7)	72 (41.1)	
**Training method** *n* = 342				0.833 ^2^
e-learning	233 (68.1)	114 (68.7)	119 (67.6)	
Face-to-face	109 (31.9)	52 (31.3)	57 (32.4)	

^1^ Mann–Whitney U Test between male group and female group; ^2^ Chi-square test between male group and female group; IQR = Interquartile range.

**Table 2:** General CPR willingness and improvement among male and female schoolchildren before and after CPR training.

	Male Group *n* (%)	Female Group *n* (%)	*p* -Value
***n* = 337**	163 (48.4)	174 (51.6)	
**General CPR willingness (baseline; t0)**			**0.011**^1,^*
Yes	145 (89.0)	169 (97.1)	
No	18 (11.0)	5 (2.9)	
**General CPR willingness (after training; t1)**			**0.038**^1,^*
Yes	156 (95.7)	172 (98.9)	
No	7 (4.3)	2 (1.1)	
**Improvement of general CPR willingness** **(after CPR training; t1)**			**0.017**^1,^*
Yes	13 (8.0)	4 (2.3)	
No	150 (92.0)	170 (97.7)	

^1^ Chi-square test between male and female group; * *p* ≤ 0.05.

**Table 3:** Influence of OHCA victim’s gender characteristics on CPR willingness among male and female schoolchildren before and after CPR training.

CPR Willingness	Male Group *n* = 166		Female Group *n* = 174		
*n* = 340	OHCA Victim Is…		OHCA Victim Is…		*p*-Value
	Female n (%)	Male n (%)	Female n (%)	Male n (%)	
Willingness to resuscitate (*t0*) (*n*;%) (yes)	101 (60.8)	121 (72.9)	147 (84.5)	120 (69.0)	0.409 ^1^ **<0.001** ^1,^*
Willingness to resuscitate (*t1*) (*n*;%) (yes)	97 (58.4)	109 (65.7)	138 (79.3)	120 (69.0)	0.318 ^1^ **<0.001** ^1,^*
Improvement in willingness to resuscitate (*t1*) (*n*;%)	36 (21.7)	28 (16.9)	19 (10.9)	33 (18.9)	0.652 ^1^ **0.006** ^1,^*

^1^ Chi-square test between male group and female group; * *p* ≤ 0.05; OHCA = Out-of-hospital cardiac arrest.

## Data Availability

The datasets used during the current study are available from the corresponding author on reasonable request.

## Abbreviations

CPR	Cardiopulmonary resuscitation
FG	Female group
MG	Male group
OHCA	Out-of-hospital cardiac arrest
